# The impact of neonatal antibiotic exposure on the development of childhood food allergies

**DOI:** 10.1007/s00431-025-06136-2

**Published:** 2025-04-21

**Authors:** Mai Ofri, Eyal Kristal, Braha Cohen, Avraham Beigelman, Guy Hazan

**Affiliations:** 1 The School of Medicine, Faculty of Health Sciences, Ben-Gurion University of the Negev, Beer-Sheva, Israel; 2 The Kipper Institute of Allergy and Immunology, Schneider Children’s Medical Center of Israel, Faculty of Medical & Health Sciences, Tel Aviv University, Tel Aviv, Israel; 3 Faculty of Medicine, Tel Aviv University, Tel Aviv, Israel; 4 The Faculty of Health Sciences, Ben-Gurion University of the Negev, Beer-Sheva, Israel; 5 Clinical Research Center, Soroka University Medical Center, Beer Sheva, Israel; 6 Pediatric Pulmonary Unit, Saban Children’s Hospital, Soroka University Medical Center, Beer Sheva, Israel

**Keywords:** Antibiotic exposure, Children, Food allergies

## Abstract

Food allergies (FAs) in children have become increasingly prevalent. While early life factors such as gut microbiome disruptions have been implicated, the association between neonatal antibiotic exposure and subsequent FAs remains a topic of ongoing debate. This nationwide cohort study aimed to investigate the impact of neonatal antibiotic exposure on the development of childhood FA. This population-based retrospective cohort study analyzed data from Clalit-Healthcare-Services, Israel’s largest state-mandated healthcare provider. The cohort included neonates (aged 0–60 days) admitted with fever between 2011 and 2018. Patients with confirmed infectious etiologies were excluded. The cohort was divided into two groups: those who received systemic antibiotics (Antibiotic ( +)) and those who did not (Antibiotic ( −)). FA cases were identified using ICD-9 codes up to age 6. Multivariate logistic regression and survival analysis models were utilized and adjusted for inflammatory markers, maternal atopy, and socioeconomic status. Among 2780 neonates, 1220 received antibiotics, while 1560 did not. The incidence of FAs was significantly higher in the Antibiotic ( +) group compared to the Antibiotic ( −) group (2.5% vs. 1.3%, *P* = 0.02). Adjusted analysis revealed that systemic antibiotic exposure during the neonatal period was associated with a threefold increased risk of FA up to age 6 (OR = 2.89, 95% CI = 1.34–6.92, *P* = 0.01).

*Conclusions*: This study provides strong evidence linking neonatal antibiotic exposure to an increased risk of childhood FAs, particularly in the first 2 years of life. The findings highlight the importance of judicious antibiotic use in young infants.

**Supplementary Information:**

The online version contains supplementary material available at 10.1007/s00431-025-06136-2.

**Table:** 

**What is Known:** *• Early-life gut microbiome disruption is linked to an increased risk of food allergies (FA).* *• Antibiotic use in infancy may contribute to FA, but the impact of neonatal antibiotics remains unclear.* **What is New:** *• In this large nationwide cohort, neonatal antibiotic exposure was associated with a nearly threefold increased risk of FA by age six, highlighting the need for careful antibiotic use in young infants.*	

## Introduction

Food allergy (FA) is a common and increasingly prevalent medical condition in the Western world, affecting an estimated 7–10% of children in North America [1]. Over recent decades, its rising prevalence has driven significant research into potential etiological factors. The implications of FAs are profound, encompassing medical risks such as anaphylaxis and other life-threatening reactions [2]. Beyond the physical health risks, FAs impose a substantial psychological burden on both affected children and their families, often leading to heightened anxiety and lifestyle limitations [3]. Additionally, the economic impact of managing FAs—ranging from medical expenses to specialized diets—is considerable, further underscoring the importance of addressing this growing public health challenge [4].

A range of multifactorial risk factors, including genetic predisposition, dietary practices, and environmental conditions influence the development of childhood food allergies [5–7]. The composition and diversity of the gut microbiome are crucial for immune regulation and have been linked to an increased risk of developing atopic diseases including FAs [8–10]. Evidence supporting the importance of normal development of the gut microbiome to prevent atopic diseases includes associations between cesarean delivery (compared to vaginal delivery), breastfeeding or maternal diet, and allergy risk in the offspring [11, 12].

It is well-established that the gut microbiome can be perturbed by numerous factors, including antibiotic treatment [13]. At the same time, evidence suggests that prenatal exposure to antibiotics may increase the risk of chronic diseases, including asthma and atopic dermatitis [14–16]. However, data regarding the association between neonatal antibiotic exposure and childhood FAs is inconclusive [17, 18]. Therefore, more data is needed to understand the associations between early-life antibiotic treatment and future FA development.

Neonates younger than 60 days of age presenting with fever are typically admitted to the hospital and assessed according to various protocols to classify their risk for bacterial infections [19, 20]. Based on these criteria, some patients undergo sepsis workups, which include blood, urine, and cerebrospinal fluid (CSF) cultures, and are treated with empiric antibiotics. Others are admitted for observation without receiving antibiotics. By leveraging a nationwide database, this study aims to compare the incidence of childhood FAs among those who received antibiotics (Antibiotic (+) group) during their hospital admission and between those who were only observed (Antibiotic (−) group), thus not receiving early-life antibiotics.

## Methods

### Study design and setting

This population-based retrospective cohort study uses a nationwide computerized database record from Clalit-Healthcare-Services (CHS). The study enrolled children born between 2011 and 2018 who were admitted to a CHS hospital for fever within the first 60 days of life. CHS is the largest state-mandated healthcare provider in Israel with over 5 million members, constituting about 50% of the population of Israel. The CHS database includes extensive demographic data, anthropometric measurements, diagnoses from community clinics and hospitals, medication dispensing information, and comprehensive laboratory data. All data were de-identified before analysis and this study involves the secondary use of already collected clinical information [21, 22].

The study received approval from the Institutional Review Board at Soroka University Medical Center (SUMC), following the Helsinki Declaration (0355–23-SOR). The need for informed consent was waived as data was retrospectively collected, and participants’ identities were kept strictly anonymous.

### Study population

The analysis encompassed all CHS members who fulfilled the above inclusion criteria, and who had a comprehensive medical history on record. Children born preterm (≤ 37 gestational weeks), small for gestational age (SGA) [23], or with chronic heart or pulmonary disease (Table S1) who had been diagnosed with neonatal fever before admission, were excluded. To focus on the effect of antibiotics and avoid potential confounding of infection that can itself disrupt the microbiome, patients with a confirmed infectious etiology were excluded. This included those who developed acute bronchiolitis or pneumonia within the first 60 days of life. Furthermore, patients with a positive sepsis workup were excluded, defined as having a positive result in one or more of the following tests conducted during their evaluation: nasal swab polymerase chain reaction (PCR) or cultures from cerebrospinal fluid (CSF), blood, or urine. Hence, this study evaluated patients aged 0–60 days presenting with fever, comparing those who received antibiotic therapy—typically intravenous ampicillin, gentamicin, and/or third-generation cephalosporins—with those who did not. Patients with confirmed infectious etiology were excluded from the analysis.

### Data sources and organization

De-identified patient-level data extracted from CHS electronic medical records (MDCLONE system) was analyzed [22]. This dataset comprised information such as date of birth, sex, ICD- 9 diagnoses of allergic rhinitis, atopic dermatitis, FAs, and data regarding birth history and family history. Information regarding the sepsis workup results in the neonate was extracted using the CHS electronic medical records, including white blood cell (WBC) count, C-reactive protein (CRP), viral PCRs, cerebral spine fluid (CSF), and blood and urine cultures. Data on maternal asthma and FA history was obtained by linking the child’s file to the mother’s chart. Socioeconomic status (SES) was included, utilizing each member’s enumeration area of residence as reported by the Israeli Central Bureau of Statistics and Points Business Mapping Ltd© 32. In the database, SES is categorized into three levels: high, medium, and low. For simplicity, SES was re-coded into a dichotomous variable: low vs. medium/high socioeconomic status.

### Study outcomes

ICD- 9 codes assigned between 0 and 6 years of age to determine the child’s diagnoses of FA primary outcome measures were utilized (refer to Table S2). Additionally, secondary outcomes including allergic rhinitis and atopic dermatitis (identified using ICD- 9 codes) were investigated.

### Statistical analysis

Initial descriptive analysis included single variable distribution, central tendency, and dispersion calculations. Further univariate analysis was conducted employing Pearson’s chi-square test for dichotomous variables, the student’s *t*-test for normally distributed continuous variables, and the Wilcoxon rank sum test for non-normally distributed continuous variables. Additionally, a univariate Kaplan–Meier analysis was conducted. Subsequently, a multivariate logistic regression model was executed while evaluating variation inflation factors. As maximum likelihood models provide point estimation of the odds ratio (OR) and a confidence interval, the Bayesian approach provides a more accurate estimation of the uncertainty around the value of interest. This is due to the method by which the OR is calculated in Bayesian statistics. To provide a better understanding of the uncertainty around the OR point value, we have implemented a Bayesian logistic regression using the *brms* package in R [18, 19]. The model used partially informative priors and provided a distribution of the possible values of the OR instead of a point estimate (and confidence intervals).

To examine the uncertainty around the OR, we have employed a Bayesian analysis to be used as a sensitivity analysis. Using the brms package in R [18, 19], the same variables as in the original logistic model for FA were entered, with brms default (partially informed) priors. This analysis enabled looking at the sampled posterior distribution of the results, accounting for the use of antibiotics in the sepsis workup, with the prior distribution of the results [24, 25]. All statistical analyses were conducted using R version 4.2.0.

## Results

**Fig. 1:**
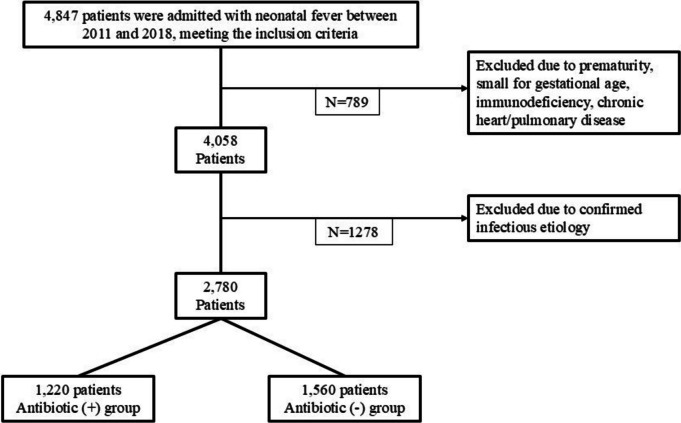
Flowchart depicting the selection process for patients included in this study. It outlines the number of premature babies born in CHS hospitals between January 1 st and December 31 st, 2011–2018 and incorporates the exclusion criteria noted in the “Methods”section

A total of 4847 infants met the initial inclusion criteria: aged 0–60 days and admitted to a CHS hospital with a fever between 2011 and 2018 (Fig. 1). Of this cohort, 789 were excluded due to underlying clinical conditions such as prematurity, small for gestational age (SGA), or chronic heart or pulmonary disease. Additionally, 421 patients were excluded due to a diagnosis of acute bronchiolitis or pneumonia within their first 60 days of life, and 857 patients with a positive sepsis workup were also excluded. In the final analysis, 2780 patients were included. These patients underwent a sepsis workup between 0 and 60 days of age without evidence of a clear infectious etiology based on laboratory results. Of this group, 1220 received systemic antibiotics (Antibiotic (+) group), while 1560 did not receive systemic antibiotics (Antibiotic (−) group).

Table 1 presents the demographic and clinical characteristics, as well as laboratory results at admission for patients included in the cohort. The Antibiotic (−) group had a larger proportion of children from higher socioeconomic status compared to the Antibiotic (+) group (61% vs. 53% in the medium or high socioeconomic status categories, respectively, *P* < 0.001). Delivery characteristics were similar between the two groups, with comparable birth weights (3298 ± 400 g vs. 3319 ± 411 g, respectively, *P* = 0.15) and cesarean delivery rates (17% vs. 18%, respectively, *P* = 0.7).

**Table 1:** Demographic and clinical characteristics and laboratory results at admission for patients included in the cohort

	Antibiotic (+) group, *N* = 1220	Antibiotic (−) group, *N* = 1560	*P*-value^***1***^
Sex (male), *n* (%)	704 (58%)	891 (57%)	0.8
Medium or High Socioeconomic status, *n* (%)	559 (53%)	867 (61%)	< 0.001
Birth weight (grams), mean ± SD	3298 ± 400	3319 ± 411	0.15
Cesarean delivery, *n* (%)	208 (17%)	274 (18%)	0.7
Multiple embryos pregnancy, *n* (%)	18 (1.5%)	27 (1.7%)	0.6
Maternal age (years), mean ± SD	29.5 ± 5.5	30.0 ± 5.2	0.03
Maternal asthma, *n* (%)	668 (55%)	909 (58%)	0.06
Maternal food allergy, *n* (%)	8 (0.7%)	21 (1.3%)	0.07
WBC (10^3^/µL), mean ± SD (IQR)	11.7 ± 5.4 (7.8, 14.6)	11.9 ± 5.8 (7.8, 14.5)	0.6
CRP (mg/dl), mean ± SD (IQR)	8 ± 21 (0, 6)	6 ± 15 (0, 4)	0.002

*WBC* white blood cells; *CRP* C-reactive protein

Maternal age was slightly higher in the Antibiotic (−) group compared to the Antibiotic (+) group (30 ± 5.2 years vs. 29.5 ± 5.5 years, respectively, *P* = 0.03), though this difference likely lacked clinical significance. There was a numerically higher proportion of family history of other atopic diseases in the Antibiotic (−) group, as reflected by maternal asthma (58% vs. 55%, *P* = 0.06) and maternal food allergy (1.3% vs. 0.7%, *P* = 0.07).

At admission, infants from both groups had similar white blood cell counts (WBC) (11.7 ± 5.4 in the Antibiotic (+) group vs. 11.9 ± 5.8 in the Antibiotic (−) group, *P* = 0.6). However, the Antibiotic (+) group had higher C-reactive protein (CRP) levels (8 ± 21 mg/dl vs. 6 ± 15 mg/dl, respectively, *P* = 0.002).

Table 2 presents a univariate analysis comparing atopic outcomes in two age groups between the Antibiotic (+) and Antibiotic (−) groups. In the Antibiotic (+) 2.5% of children had FAs compared to 1.3% in the Antibiotic (−) group (*P* = 0.02). The OR for FA in the Antibiotic (+) group vs. the Antibiotic (−) group was 1.94, (95% CI, 1.10–3.48; *P*-value = 0.023). A slightly higher rate of atopic dermatitis was observed in the Antibiotic (−) group compared to the Antibiotic (+) group (11% vs. 9.4%; *P* = 0.3; OR 0.3; 95% CI, 0.68–1.13; *P*-value = 0.3). The proportions of allergic rhinitis were similar in the Antibiotic (+) and Antibiotic (−) groups (2.6% vs. 3.2%, P = 0.4, respectively, OR 0.81, 95% CI 0.51–1.27, *P*-value = 0.4).

**Table 2:** Univariate analysis comparing atopic outcomes in two age groups between the Antibiotic (+) and Antibiotic (**−**) groups

Characteristic	Antibiotic (+) group, *N* = 1220	Antibiotic (−) group, *N* = 1560	OR^1^	5%, 95% CI	*P*-value
The proportion of food allergy diagnoses (*n*, %)	30 (2.5%)	20 (1.3%)	1.94	1.10, 3.48	0.02
The proportion of atopic dermatitis diagnoses (*n*, %)	115 (9.4%)	165 (11%)	0.88	0.68, 1.13	0.3
The proportion of allergic rhinitis diagnoses (*n*, %)	32 (2.6%)	50 (3.2%)	0.81	0.51, 1.27	0.4

^1^*OR* odds ratio, *CI* confidence Interval

**Fig. 2:**
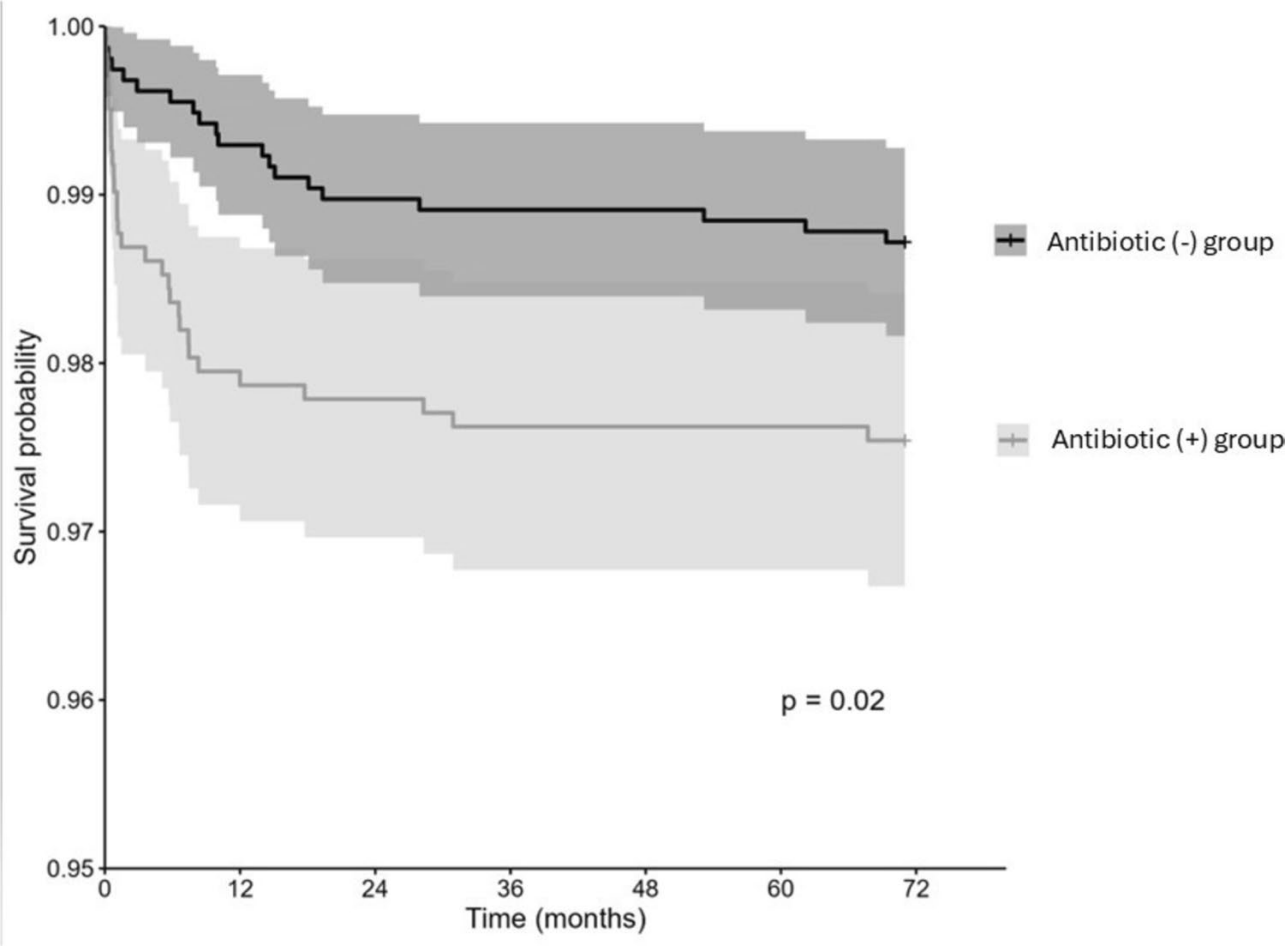
Kaplan–Meier survival analysis for the development of food allergy (FA) between birth and 6 years of age, comparing children with antibiotic exposure (antibiotic [+] group) and without antibiotic exposure (antibiotic [−] group). The *P*-value was calculated using the log-rank test (*p* = 0.02). Shaded areas represent the 95% confidence intervals

To assess whether age influences the association between antibiotic exposure and FA, a univariate survival analysis was conducted. As shown in Fig. 2, a statistically significant difference was observed between the Antibiotic (+) and Antibiotic (−) groups (*P* = 0.02). Notably, the primary effect of antibiotic exposure on the difference between the groups occurred within the first 2 years of life.

Next, multivariate analyses were conducted (Table 3). The association between the administration of systemic antibiotics during the neonatal period and the diagnosis of FA was analyzed, adjusting for inflammatory parameters at presentation (CRP levels at admission), maternal atopy history (maternal asthma or food allergy), and SES. The adjusted OR for antibiotic use was positively associated with the incidence of FA, with the rate of FA being 2.89 times higher in the Antibiotic (+) group compared to the Antibiotic (−) group (95% CI, 1.34–6.92, *P* = 0.01).

**Table 3:** Multivariate logistic regression analysis of factors associated with diagnosing food allergy in children aged 0–6 years

	FA (+) *N* = 50	FA (−) *N* = 2730	OR	95% CI^*1*^	*P*-value
Antibiotic (+)	**30 (60%)**	**1190 (44%)**	**2.89**	**1.34, 6.92**	**0.01**
CRP (mg/dl) at admission (median, IQR)	4 (1, 11)	1 (0, 5)	1.01	1.00, 1.02	0.02
Medium/higher socioeconomic status	16 (37%)	1410 (58%)	0.42	0.19, 0.87	0.02
Maternal asthma	23 (46%)	1554 (57%)	0.53	0.26, 1.08	0.08
Maternal food allergy	1 (2%)	28 (1%)	5.06	0.27, 27.9	0.13

The non-bolded items are the variables for which Antibiotic (+) was adjusted in the multivariate model

*IQR* interquartile range

**Fig. 3:**
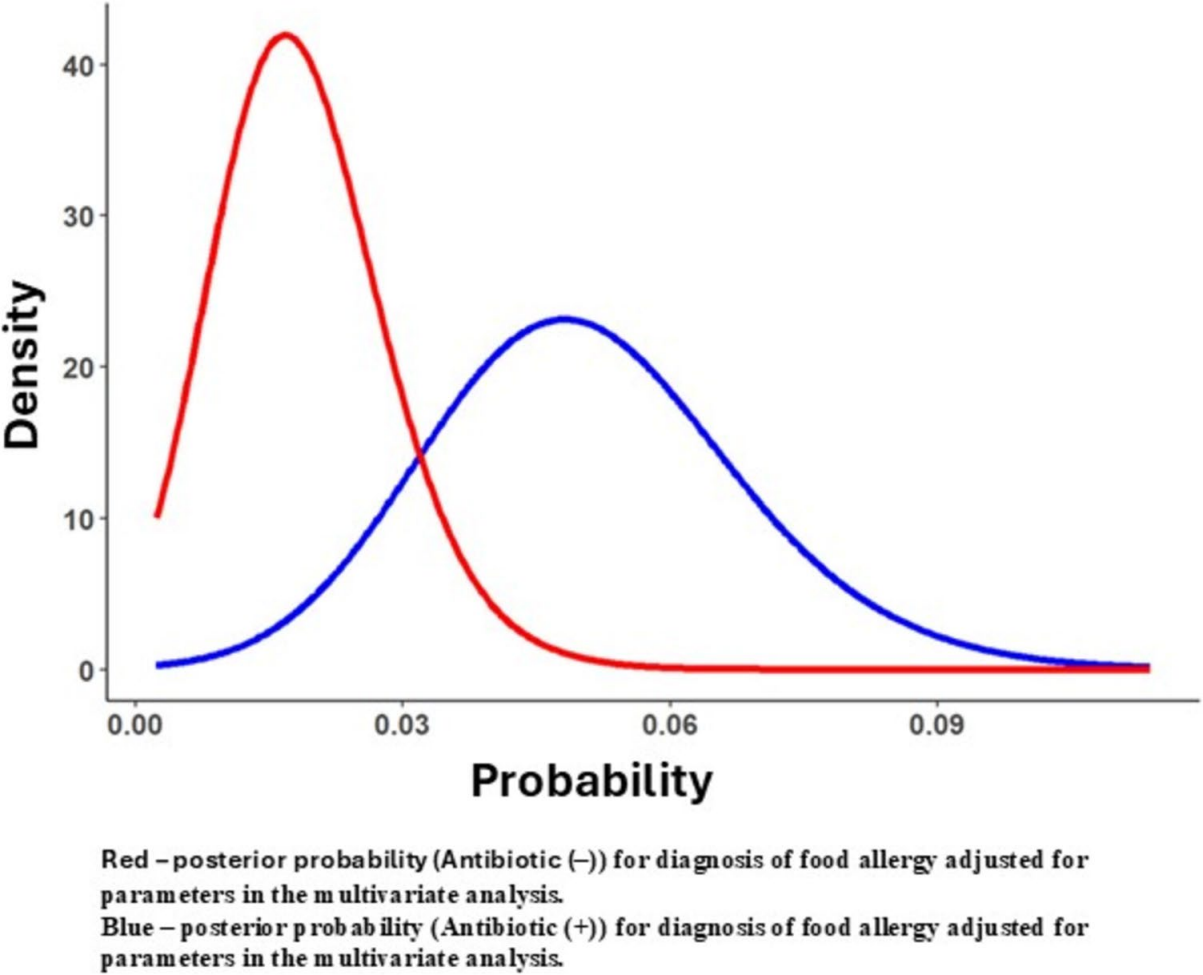
Bayesian analysis of multivariate logistic regression examining the association between antibiotic administration and food allergy diagnosis up to 6 years of age. Comparison between the Antibiotic (+) group (blue) and the Antibiotic (−) group (Red)

A sensitivity analysis using a Bayesian approach was conducted to evaluate the diagnosis of FA up to 6 years of age (Fig. 3). This analysis employed a Bayesian logistic regression (with similar variables to the previously described model, Table 3). The results portray the posterior distribution of FA for each of the antibiotic groups (Antibiotic (+) in blue vs. Antibiotic (−) in red Fig. 3). These results are consistent with the findings from the logistic regression, showing approximately three times higher rate of FA in children treated with antibiotics during the neonatal period compared to those who did not receive systemic antibiotic treatment.

## Discussion

This nationwide retrospective cohort study identified a significant association between neonatal exposure to systemic antibiotics and approximately a threefold increase in the prevalence of childhood food allergies (OR = 2.89, 95% CI = 1.34–6.92, *P* = 0.01). These findings were consistent across univariate analysis, multivariate logistic regression, and sensitivity analyses using a Bayesian approach, with adjustments for inflammatory markers at admission, maternal atopy, and family socioeconomic status. The majority of the impact occurs within the first 2 years of life. This aligns with the proposed mechanism involving gut microbiota and immune system development, supporting the idea that neonatal antibiotic exposure primarily influences early-onset FAs.

There is growing evidence of the association between early-life antibiotic exposure and food allergy in the literature, yet, the potential confounding effect of infection (for which the antibiotics was prescribed) had not fully been accounted for [26]. A large-scale study on Medicaid patients found a strong association between antibiotic prescriptions during the first year of life and food allergy [27], while a nationwide South Korean study reported a 5% increased risk of food allergy associated with antibiotic use during the first six months of life [18]. Notably, most studies have focused on antibiotic prescriptions during infancy (up to six months of age) rather than the neonatal period, often facing limitations such as small sample sizes and insufficient adjustment for genetic factors, including maternal food allergies or asthma [28, 29].

Several factors may modify the relationship between neonatal antibiotic exposure and food allergies. The mode of delivery, for instance, can independently influence the composition of the neonatal microbiome, potentially interacting with the effects of antibiotics [13]. A plausible mechanism underlying this association is the disruption of the neonatal gut microbiome [30, 31]. Early life, particularly the neonatal period, is critical for the establishment of a healthy microbiome, which plays a central role in immune system maturation [13, 32]. Systemic antibiotic use during this time can disturb the gut microbiota, reducing microbial diversity and altering the balance of beneficial bacteria [13]. It is caused by several mechanisms including reduction of microbial diversity, altering gut composition, and impairing the production of essential metabolites [33]. This disruption can impair regulatory T-cell function, increase gut permeability, and lead to immune dysregulation, raising the risk of allergies and autoimmune diseases [33]. This dysbiosis may hinder the development of oral tolerance to dietary antigens, potentially increasing the risk of food allergies. Genetic predisposition, including maternal food allergies or asthma, may also play a significant role [34]. Additionally, disentangling the effects of the antibiotics themselves from those of the underlying infections for which they were prescribed remains challenging. Socioeconomic status was also found to be associated with the likelihood of food allergies, with lower socioeconomic status linked to a higher risk of developing food allergies [35]. This relationship was evident in the results of this study, prompting an adjustment for this variable in the analysis.

The rate of food allergy in this cohort was approximately 2%, which is much lower than the reported prevalence of childhood food allergies in North America and Europe, which was estimated as 8–10% [1]. However, the food allergy prevalence detected in our study is very similar to the prevalence previously reported in Israel [36]. The lower prevalence of food allergy in Israel compared to North America is likely related to the earlier introduction of allergenic foods [37] but may be related to other environmental factors. Nevertheless, the detected food allergy prevalence in our cohort suggests that the cohort is representative of the general population [38].

This is a large study, including the majority of hospitalized infants, leveraging the comprehensive and universal EHRs used in the CHS. To minimize confounding factors, we selected a control group of neonates admitted with fever, assuming an infectious etiology even when not explicitly identified. This approach helped mitigate the potential influence of the underlying infection. Additionally, we accounted for baseline differences in immune activation by adjusting for inflammatory markers. Notably, the antibiotic-treated group exhibited higher CRP levels compared to the untreated group; a clinically reasonable finding that likely explains the decision to administer antibiotics at the time of admission. By addressing these complexities, our study provides a more refined understanding of the relationship between neonatal antibiotic exposure and the subsequent risk of developing food allergies.

A key strength of this study is the ability to link maternal records, allowing for the identification of diagnoses indicative of genetic atopic predisposition. Furthermore, unlike many previous studies that relied on antibiotic prescriptions, which do not always reflect actual usage, this study focused exclusively on hospitalized patients, enhancing the accuracy of antibiotic exposure assessment. By recruiting a control group of hospitalized neonates with fever, the study also effectively adjusted for factors such as early-life infections and their severity, as both groups shared similar clinical contexts of hospitalization.

However, this study has several limitations. A major limitation of this study is its cross-sectional design, which prevents the establishment of causality and only allows for the identification of associations. Its retrospective design relies on ICD- 9 codes, which may underreport the true incidence of diagnoses. Although this method of identifying FAs has been used in large database studies [39, 40], relying on ICD- 9 codes may result in misclassifying true IgE-mediated food allergies. Furthermore, diagnoses made by private physicians may not be recorded in real-time or may be missing from the electronic medical system altogether. This issue is more pronounced for conditions like allergic rhinitis, mild atopic dermatitis, and asthma in patients who do not receive preventive treatment, which may be documented less consistently by physicians due to less pronounced clinical symptoms. In contrast, the clinical significance of food allergies likely ensures more consistent documentation, reducing the extent of underreporting for this specific condition. Furthermore, atopic dermatitis may be both a potential outcome of antibiotic use and a contributing factor to the development of food allergies. This complex interplay makes it challenging to distinguish between cause and effect [41]. Additionally, due to the nature of big-data studies, detailed information on individual patient skin prick tests, food challenges, or dietary and environmental exposures was unavailable.This study provides strong evidence of an association between neonatal antibiotic exposure and an increased risk of childhood food allergies, with a threefold higher prevalence in those exposed to systemic antibiotics. The findings highlight the importance of judicious antibiotic use in neonates and emphasize the need for further prospective research into the mechanisms underlying the association between antibiotic use in the neonatal period and food allergies in childhood, as well as strategies for preventing gut microbiome disruption following such exposure.

## Supplementary Information

Below is the link to the electronic supplementary material.

Supplementary file1 (DOCX 24 KB)

## Data Availability

The data used in this study are not publicly available due to Clalit-Helathcare Services policy. However, access may be granted upon reasonable request to the correspondng author.

## Abbreviations

CHS: Clalit healthcare services
CRP: C-reactive protein
CSF: Cerebrospinal fluid
FA: Food allergy
OR: Odds ratio
PCR: Polymerase chain reaction
SES: Socioeconomic status
SGA: Small for gestational age
SUMC: Soroka University Medical Center
WBC: White blood count

## References

[1] Gupta RS, Warren CM, Smith BM et al (2018) The public health impact of parent-reported childhood food allergies in the United States. Pediatrics 142(6). 10.1542/peds.2018-1235 10.1542/peds.2018-1235 PMC6317772 30455345

[2] Warren CM, Sehgal S, Sicherer SH, Gupta RS (2024) Epidemiology and the growing epidemic of food allergy in children and adults across the globe. Curr Allergy Asthma Rep 24(3):95–106. 10.1007/s11882-023-01120-y 38214821 10.1007/s11882-023-01120-y

[3] Bingemann TA, LeBovidge J, Bartnikas L, Protudjer JLP, Herbert LJ (2024) Psychosocial impact of food allergy on children and adults and practical interventions. Curr Allergy Asthma Rep 24(3):107–119. 10.1007/s11882-023-01121-x 38261244 10.1007/s11882-023-01121-x PMC11340266

[4] Warren C, Bartell T, Nimmagadda SR, Bilaver LA, Koplin J, Gupta RS (2022) Socioeconomic determinants of food allergy burden: a clinical introduction. Ann Allergy Asthma Immunol 129(4):407–416. 10.1016/j.anai.2022.07.021 35914663 10.1016/j.anai.2022.07.021

[5] Di Costanzo M, De Paulis N, Capra ME, Biasucci G (2022) Nutrition during pregnancy and lactation: epigenetic effects on infants’ immune system in food allergy. Nutrients 14(9). 10.3390/nu14091766 10.3390/nu14091766 PMC9103859 35565735

[6] Hong X, Tsai HJ, Wang X (2009) Genetics of food allergy. Curr Opin Pediatr 21(6):770–776. 10.1097/MOP.0b013e32833252dc 19851108 10.1097/MOP.0b013e32833252dc PMC2892276

[7] Rennie GH, Zhao J, Camus-Ela M et al (2023) Influence of lifestyle and dietary habits on the prevalence of food allergies: a scoping review. Foods 12(17). 10.3390/foods12173290 10.3390/foods12173290 PMC10486777 37685223

[8] Pantazi AC, Mihai CM, Balasa AL et al (2023) Relationship between gut microbiota and allergies in children: a literature review. Nutrients 15(11). 10.3390/nu15112529 10.3390/nu15112529 PMC10255222 37299492

[9] Shao T, Hsu R, Rafizadeh DL et al (2023) The gut ecosystem and immune tolerance. J Autoimmun 141:103114. 10.1016/j.jaut.2023.103114 37748979 10.1016/j.jaut.2023.103114

[10] Stefka AT, Feehley T, Tripathi P et al (2014) Commensal bacteria protect against food allergen sensitization. Proc Natl Acad Sci U S A 111(36):13145–13150. 10.1073/pnas.1412008111 25157157 10.1073/pnas.1412008111 PMC4246970

[11] Liu X, Zhou J, Chen J et al (2024) Risk of asthma and allergies in children delivered by cesarean section: a comprehensive systematic review. J Allergy Clin Immunol Pract. 10.1016/j.jaip.2024.06.022 10.1016/j.jaip.2024.06.022 38908434

[12] Abrams EM, Shaker MS, Chan ES, Brough HA, Greenhawt M (2023) Prevention of food allergy in infancy: the role of maternal interventions and exposures during pregnancy and lactation. Lancet Child Adolesc Health 7(5):358–366. 10.1016/S2352-4642(22)00349-2 36871575 10.1016/S2352-4642(22)00349-2

[13] Patangia DV, Anthony Ryan C, Dempsey E, Paul Ross R, Stanton C (2022) Impact of antibiotics on the human microbiome and consequences for host health. Microbiologyopen 11(1):e1260. 10.1002/mbo3.1260 35212478 10.1002/mbo3.1260 PMC8756738

[14] Loewen K, Monchka B, Mahmud SM, t Jong G, Azad MB (2018) Prenatal antibiotic exposure and childhood asthma: a population-based study. Eur Respir J 52(1). 10.1183/13993003.02070-2017 10.1183/13993003.02070-2017 29678946

[15] Ortqvist AK, Lundholm C, Kieler H et al (2014) Antibiotics in fetal and early life and subsequent childhood asthma: nationwide population based study with sibling analysis. BMJ 349:g6979. 10.1136/bmj.g6979 25432937 10.1136/bmj.g6979 PMC4247260

[16] Stokholm J, Sevelsted A, Bonnelykke K, Bisgaard H (2014) Maternal propensity for infections and risk of childhood asthma: a registry-based cohort study. Lancet Respir Med 2(8):631–637. 10.1016/S2213-2600(14)70152-3 25066330 10.1016/S2213-2600(14)70152-3

[17] Kamphorst K, Vlieger AM, Oosterloo BC, Waarlo S, van Elburg RM (2021) Higher risk of allergies at 4–6 years of age after systemic antibiotics in the first week of life. Allergy 76(8):2599–2602. 10.1111/all.14829 33772817 10.1111/all.14829

[18] Oh J, Lee M, Park J et al (2024) Prenatal and postnatal exposure to antibiotics and risk of food allergy in the offspring: a nationwide birth cohort study in South Korea. Pediatr Allergy Immunol 35(3):e14114. 10.1111/pai.14114 38529692 10.1111/pai.14114

[19] Jaskiewicz JA, McCarthy CA, Richardson AC et al (1994) Febrile infants at low risk for serious bacterial infection–an appraisal of the Rochester criteria and implications for management. Febrile infant collaborative study group. Pediatrics 94(3):390–396 8065869

[20] Pantell RH, Roberts KB, Adams WG et al (2021) Evaluation and management of well-appearing febrile infants 8 to 60 days old. Pediatrics 148(2). 10.1542/peds.2021-052228 10.1542/peds.2021-052228 34281996

[21] Amram T, Duek OA, Golan-Tripto I, Goldbart A, Greenberg D, Hazan G (2025) The interplay between respiratory syncytial virus and asthma inception: insights gained from the COVID-19 pandemic. Pediatr Pulmonol 60:e27474. 10.1002/ppul.27474 39760467 10.1002/ppul.27474 PMC11748096

[22] Hasson HO, Bachar Y, Hazan I et al (2024) The impact of palivizumab for respiratory syncytial virus prophylaxis on preschool childhood asthma. Vaccines (Basel) 12(11). 10.3390/vaccines12111269 10.3390/vaccines12111269 PMC11598595 39591172

[23] Dollberg S, Haklai Z, Mimouni FB, Gorfein I, Gordon ES (2005) Birth weight standards in the live-born population in Israel. Isr Med Assoc J 7(5):311–314 15909464

[24] Burkner PC, Charpentier E (2020) Modelling monotonic effects of ordinal predictors in Bayesian regression models. Br J Math Stat Psychol 73(3):420–451. 10.1111/bmsp.12195 31943157 10.1111/bmsp.12195

[25] Nalborczyk L, Batailler C, Loevenbruck H, Vilain A, Burkner PC (2019) An introduction to Bayesian multilevel models using brms: a case study of gender effects on vowel variability in standard Indonesian. J Speech Lang Hear Res 62(5):1225–1242. 10.1044/2018_JSLHR-S-18-0006 31082309 10.1044/2018_JSLHR-S-18-0006

[26] Netea SA, Messina NL, Curtis N (2019) Early-life antibiotic exposure and childhood food allergy: a systematic review. J Allergy Clin Immunol 144(5):1445–1448. 10.1016/j.jaci.2019.08.001 31415783 10.1016/j.jaci.2019.08.001

[27] Li M, Lu ZK, Amrol DJ et al (2019) Antibiotic exposure and the risk of food allergy: Evidence in the US Medicaid pediatric population. J Allergy Clin Immunol Pract 7(2):492–499. 10.1016/j.jaip.2018.09.036 30468878 10.1016/j.jaip.2018.09.036

[28] Hirsch AG, Pollak J, Glass TA et al (2017) Early-life antibiotic use and subsequent diagnosis of food allergy and allergic diseases. Clin Exp Allergy 47(2):236–244. 10.1111/cea.12807 27562571 10.1111/cea.12807 PMC5345106

[29] Risnes KR, Belanger K, Murk W, Bracken MB (2011) Antibiotic exposure by 6 months and asthma and allergy at 6 years: findings in a cohort of 1,401 US children. Am J Epidemiol 173(3):310–318. 10.1093/aje/kwq400 21190986 10.1093/aje/kwq400 PMC3105273

[30] Bunyavanich S, Berin MC (2019) Food allergy and the microbiome: current understandings and future directions. J Allergy Clin Immunol 144(6):1468–1477. 10.1016/j.jaci.2019.10.019 31812181 10.1016/j.jaci.2019.10.019 PMC6905201

[31] Huang H, Jiang J, Wang X, Jiang K, Cao H (2024) Exposure to prescribed medication in early life and impacts on gut microbiota and disease development. EClinicalMedicine 68:102428. 10.1016/j.eclinm.2024.102428 38312240 10.1016/j.eclinm.2024.102428 PMC10835216

[32] Park H, Park NY, Koh A (2023) Scarring the early-life microbiome: its potential life-long effects on human health and diseases. BMB Rep 56(9):469–481. 10.5483/BMBRep.2023-0114 37605613 10.5483/BMBRep.2023-0114 PMC10547969

[33] Takiishi T, Fenero CIM, Camara NOS (2017) Intestinal barrier and gut microbiota: shaping our immune responses throughout life. Tissue Barriers 5(4):e1373208. 10.1080/21688370.2017.1373208 28956703 10.1080/21688370.2017.1373208 PMC5788425

[34] Fujimura T, Lum SZC, Nagata Y, Kawamoto S, Oyoshi MK (2019) Influences of maternal factors over offspring allergies and the application for food allergy. Front Immunol 10:1933. 10.3389/fimmu.2019.01933 31507589 10.3389/fimmu.2019.01933 PMC6716146

[35] Davis CM, Apter AJ, Casillas A et al (2021) Health disparities in allergic and immunologic conditions in racial and ethnic underserved populations: a work group report of the AAAAI committee on the underserved. J Allergy Clin Immunol 147(5):1579–1593. 10.1016/j.jaci.2021.02.034 33713767 10.1016/j.jaci.2021.02.034

[36] Garkaby J, Epov L, Musallam N et al (2021) The sesame-peanut conundrum in israel: reevaluation of food allergy prevalence in young children. J Allergy Clin Immunol Pract 9(1):200–205. 10.1016/j.jaip.2020.08.010 32822919 10.1016/j.jaip.2020.08.010

[37] Du Toit G, Katz Y, Sasieni P et al (2008) Early consumption of peanuts in infancy is associated with a low prevalence of peanut allergy. J Allergy Clin Immunol 122(5):984–991. 10.1016/j.jaci.2008.08.039 19000582 10.1016/j.jaci.2008.08.039

[38] Sicherer SH, Warren CM, Dant C, Gupta RS, Nadeau KC (2020) Food allergy from infancy through adulthood. J Allergy Clin Immunol Pract 8(6):1854–1864. 10.1016/j.jaip.2020.02.010 32499034 10.1016/j.jaip.2020.02.010 PMC7899184

[39] Clark S, Gaeta TJ, Kamarthi GS, Camargo CA (2006) ICD-9-CM coding of emergency department visits for food and insect sting allergy. Ann Epidemiol 16(9):696–700. 10.1016/j.annepidem.2005.12.003 16516491 10.1016/j.annepidem.2005.12.003

[40] Hill DA, Grundmeier RW, Ram G, Spergel JM (2016) The epidemiologic characteristics of healthcare provider-diagnosed eczema, asthma, allergic rhinitis, and food allergy in children: a retrospective cohort study. BMC Pediatr 16:133. 10.1186/s12887-016-0673-z 27542726 10.1186/s12887-016-0673-z PMC4992234

[41] Brough HA, Nadeau KC, Sindher SB et al (2020) Epicutaneous sensitization in the development of food allergy: what is the evidence and how can this be prevented? Allergy 75(9):2185–2205. 10.1111/all.14304 32249942 10.1111/all.14304 PMC7494573

