# Supplementary material for: The impact of neonatal antibiotic exposure on the development of childhood food allergies
Source: Eur J Pediatr. 2025 Apr 21;184(5):304. doi: 10.1007/s00431-025-06136-2 (PMC12011889; doi:10.1007/s00431-025-06136-2)
Supplement: Supplementary file 1 — Supplementary file1 (DOCX 24 KB) [file 431_2025_6136_MOESM1_ESM.docx]

**Supplementary:**

**Table 1S: ICD-9 codes of chronic pulmonary/heart diseases:**

| **Diagnosis** | **ICD-9 code** |
| --- | --- |
| Chronic respiratory disease of newborn | 770.7, 770.8 |
| Cystic Fibrosis | 277.02, 277.00, 277.09 |
| Truncus arteriosus, Transposition of great vessels, Tetralogy of Fallot, Common ventricle (Single ventricle), Ventricular septal defect, Ostium secundum type atrial septal defect (ASD),Endocardial cushion defects (Atrioventricular canal defect), cor triatriatum, Other specified defects of septal closure,Anomalies of pulmonary valve (e.g., pulmonary stenosis), Tricuspid valve atresia and stenosis, Ebstein's anomaly, Congenital stenosis of aortic valve, Congenital insufficiency of aortic valve, Congenital mitral stenosis, Congenital mitral insufficiency, Hypoplastic left heart syndrome, Congenital subaortic stenosis, Infundibular pulmonic stenosis, Obstructive defects of heart, other, Coronary artery anomalies, Congenital heart block, Congenital heart aneurysm, Other specified congenital anomalies of heart, Congenital anomaly of heart, unspecified, Patent ductus arteriosus (PDA), Coarctation of aorta, Interrupted aortic arch, Other anomalies of pulmonary artery, Anomalous pulmonary venous connection, Total anomalous pulmonary venous connection (TAPVC), Other specified anomalies of pulmonary artery, Other anomalies of great veins, Congenital anomaly of circulatory system, unspecified, Anomalies of coronary vessels, Arteriovenous malformation | 745.x, 746.x, 747.x |

**Table 2S: ICD-9 code of atopic diseases**

| **Diagnosis** | **ICD-9 code** |
| --- | --- |
| Food allergy | V15.0[1-5], 995.6x |
| Atopic dermatitis | 691.x |
| Allergic rhinitis | 477.x |
